# Reproductive toxicity and meiotic dysfunction following exposure to the pesticides Maneb, Diazinon and Fenarimol†
†Electronic supplementary information (ESI) available. See DOI: 10.1039/c4tx00141a



**DOI:** 10.1039/c4tx00141a

**Published:** 2015-02-02

**Authors:** Parodi Daniela A, Sjarif Jasmine, Chen Yichang, Allard Patrick

**Affiliations:** a Institute for Society and Genetics , University of California , Los Angeles , Los Angeles , USA . Email: pallard@ucla.edu ; Tel: +1 310 825-5257; b Department of Environmental Health Sciences , University of California , Los Angeles , Los Angeles , USA; c Molecular Toxicology Inter-Departmental Program , University of California , Los Angeles , Los Angeles , USA

## Abstract

The comprehensive identification and mechanistic analysis of reproductive toxicants constitutes one of the major hurdles in the toxicological assessment of chemicals originating from the large number of chemicals to be tested and the difficulty in examining germ cells at various stages of their development. We previously described the development of an assay in the roundworm *Caenorhabditis elegans* that allows the detection of chemicals bearing aneugenic activity and that could be used for the detection of germline toxicity. We present here new evidence for the reproductive toxicity of three pesticides identified in our germline toxicity assay: Maneb, Diazinon and Fenarimol. We show that all three pesticides cause an acute germline nuclear loss in exposed nematodes in a dose-dependent fashion. The loss of germline nuclei coincides with the meiotic stage of pachytene during Prophase I and is dependent on the germline apoptotic machinery suggesting activation of a meiotic checkpoint. Further investigation revealed a profound dysregulation of the meiotic program as evidenced by (1) an alteration of the kinetics of double strand repair, (2) the disruption of the process of chromosome morphogenesis at the end of Prophase I and (3) the reorganization of the meiotic differentiation gradient inherent to the *C. elegans* germline following exposure to Maneb and Diazinon. These defects correlate with a significant increase in embryonic lethality and a corresponding decrease in the number of progeny. These results therefore provide strong evidence for the reproductive toxicity of Maneb, Diazinon and Fenarimol rooted in the alteration of early steps of germ cell differentiation.

## Abstract 2

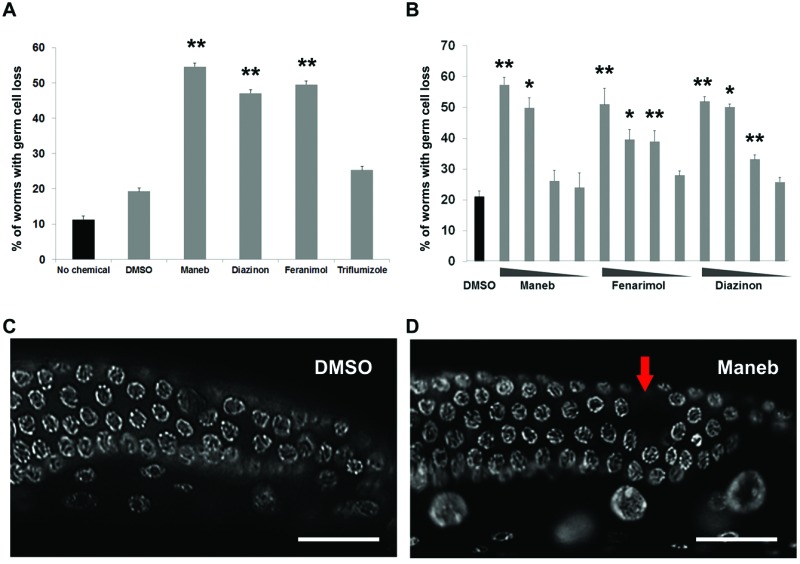
The comprehensive identification and mechanistic analysis of reproductive toxicants constitutes one of the major hurdles in the toxicological assessment of chemicals.

## Introduction

While there is mounting evidence that environmental chemical exposures can affect normal reproductive processes and chromosomal segregation,^1^ we are still limited in our ability to quickly identify which of the chemicals introduced into our environment are interfering with fertility. This is due in part to the staggering number of chemicals currently in use worldwide, totaled at 100 000, as well as the difficulty in examining the early stages of the meiotic process which take place during embryonic development in female mammals.

To address these issues, we previously reported the development and validation of a high-throughput screening platform to test reproductive toxicity in the roundworm *Caenorhabditis elegans*.^2^ *C. elegans* is a commonly used genetic model system which offers great advantages for the study of reproductive toxicity such as conservation of key reproductive and meiotic pathways, transparency, a high proportion of germ cells and genetic tractability.^3^ The reproductive toxicity assay we have developed in the nematode is based on the expression of a GFP-tagged reporter, termed *Pxol-1::GFP*, which is specifically expressed in embryonic cells that inherit one X chromosome instead of the normal two X state.^4^ As perturbation of normal germline function frequently leads to chromosome segregation errors, we hypothesized that the *C. elegans Pxol-1::GFP* reporter strain could be used to detect chemicals that act as germline toxicants and therefore perturb normal chromosome segregation. We tested this hypothesis by screening a panel of approximately 50 chemicals, mainly pesticides, from the ToxCast Phase I library,^5^ and successfully identified known mammalian aneugens as well as previously un-characterized chemicals. Here, we focused our study on three of the strongest hits from the assay: Diazinon, Fenarimol and Maneb which induced aneuploidy embryos 27.5-fold, 12.1-fold and 4.9-fold over DMSO respectively following a 65-hour exposure.^2^

Maneb is a fungicide related to the thiocarbamate pesticide family and is used on a wide variety of fruits, vegetables and field crops. Maneb has been shown to interfere with hormonal glucocorticoid signaling by interfering with the enzyme 11β-hydroxysteroid dehydrogenase type 2.^6^ Diazinon is an organophosphate insecticide used for pest control in the agricultural industry and mainly functions as an acetylcholinesterase inhibitor.^7^ Finally, Fenarimol is an organochlorine fungicide that interferes with the fungal sterol biosynthesis pathway.^8^ Interestingly, some, but scarce, evidence suggests that all three may carry aneugenic and clastogenic activities in cell culture settings or rodent models although no impacted germline pathways have been identified to date.^9^ The research presented here supports the aneugenicity of Maneb, Diazinon and Fenarimol by providing a mechanistic examination of their action on the germline.

## Results and discussion

### Loss of germline nuclei following exposure to Maneb, Diazinon and Fenarimol

**Fig. 1:**
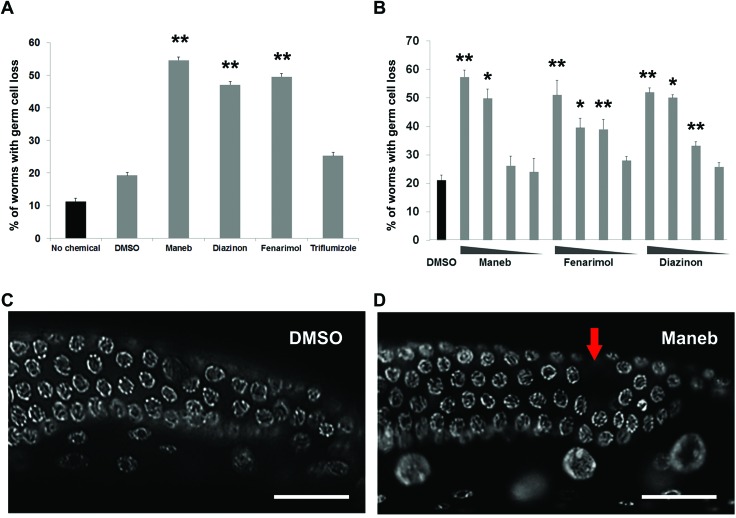
Exposure to Maneb, Diazinon and Fenarimol results in germline cell loss. (A, B) Percentage of worms with germ cell loss in worms exposed to no chemical, vehicle (0.1% DMSO) or pesticides at 100 μM for 24 hours. Error bars (SEM) represent the comparison between DMSO and the pesticide exposed groups. *n* = 182–132, *p ≤ 0.05 and **p ≤ 0.005. (B) Dose–response of the three positive pesticides showing the percentage of worms with germ cell loss in worms exposed either with vehicle (0.1% DMSO) or pesticides at the following doses in order: 100 μM, 10 μM, 1 μM, 0.1 μM. *n* = 87–43, *p ≤ 0.05 and **p ≤ 0.005. (C–D) Images of DAPI-stained whole-mount gonads from age-matched hermaphrodites exposed to either (C) DMSO or (D) Maneb at 100 μM. Scale bar, 20 μm. Red arrow indicates the location of the nuclear gap.

We first examined the germline of the *C. elegans* worms exposed to the pesticides by DAPI staining. The most obvious phenotype observed for exposure to all three chemicals was the presence of a significant loss of germline nuclei which can be visualized as gaps, defined as an empty area corresponding to the size of two nuclei or more, within an otherwise even field of nuclei (Fig. 1C, 1D). The penetrance of the phenotype for a exposure of 100 μM for 24 hours was relatively high with a frequency ranging from 45% for Diazinon to 54% for Maneb compared to 19% for DMSO and 11% for buffer-only (no chemical) exposures (Fig. 1A). For Maneb, Diazinon and Fenarimol, the increase in germline with overt nuclear loss was highly statistically significant (*p* ≤ 0.005). By contrast, another pesticide tested, the fungicide Triflumizole did not show a significant increase compared to controls (*p* = 0.09). Importantly, beside their germline phenotypes, no overt signs of toxicity were observed including changes in morphology, locomotion or behavior following exposure to any of the chemicals.

We established the dose–response for each of the three positive pesticides: Maneb, Diazinon and Fenarimol by exposing the worms to a range of concentrations: 100 nM, 1 μM, 10 μM and 100 μM. The 100 μM dose is commonly used for chemical screens in *C. elegans* and screening outcomes at that concentration has been shown to correlate well with mammalian reproductive endpoints.^10–12^ The concentration range used here therefore represents a large three log interval. For all 3 chemicals, we observed a dose–dependent relationship with regards to the percentage of worms displaying acute germline nuclear loss (Fig. 1B). For Maneb, 100 μM and 10 μM were significantly higher than DMSO control while for Diazinon and Fenarimol, 100 μM, 10 μM and 1 μM all led to levels of gaps that were significantly elevated. Therefore, these results indicate that exposure to Maneb, Diazinon and Fenarimol leads to significant nuclear loss in the germline in a dose–dependent manner.

### Pesticide exposure induces germline apoptosis and pachytene checkpoint activation

**Fig. 2:**
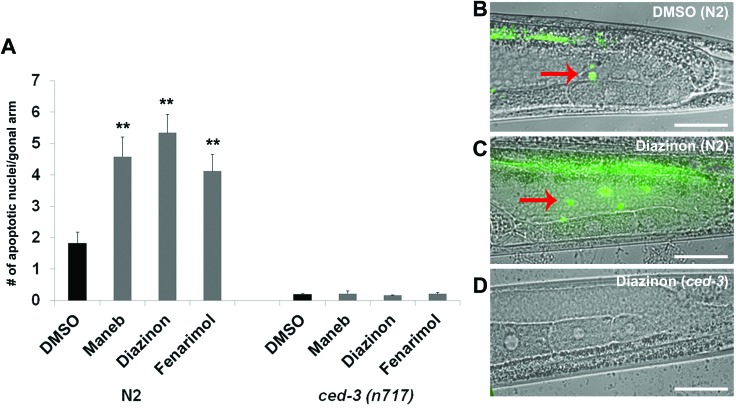
Exposure to Maneb, Diazinon and Fenarimol results in an increase in apoptosis in the germline. (A) Quantitation of germ cell apoptosis in wild type N2 and *ced-3(n717)* worms exposed either with vehicle (0.1% DMSO) or pesticides (100 μM) for 24 hours. Loss function of *ced-3* blocks germ cell apoptosis induced by the exposure to pesticides. Error bars represent SEM. *n* = 105–84, **p ≤ 0.005. (B–D) Representative pictures of germ cell apoptosis. Acridine Orange staining of gonads from age-matched hermaphrodites exposed to either (B) DMSO or (C–D) a representative pesticide (Diazinon) in (C) wild type N2 or (D) *ced-3(n717)* worms. Bright AO staining (red arrows) was observed in the apoptotic germ cells of the wild-type N2 worms. The scale bar represents 20 μm.

The location of the gaps caused by Maneb, Diazinon and Fenarimol exposure coincides with the stage of late pachytene, where germline nuclei are subject to two distinct checkpoints: the DNA damage, p53 (*Cep-1*)-dependent checkpoint and the homologous chromosome synapsis, PCH-2 mediated checkpoint.^13–15^ As activation of either checkpoint culminates in induction of apoptosis, we monitored the number of nuclei undergoing cell death in the germlines of the treated animals by staining the germline with acridine orange, a commonly used stain for cell death in *C. elegans* and a variety of other models.^16^ Consistent with the incidence of nuclear loss, we observed elevated acridine orange staining in late pachytene in the germline of worms exposed to 100 μM of either Maneb, Diazinon or Fenarimol (*p* ≤ 0.005, Fig. 2) while again Triflumizole did not show a significant increase (see Supplemental Fig. S1†). Thus, for all subsequent experiments, we compared the effect of Maneb, Diazinon and Fenarimol and concluded that Triflumizole was likely to not be a reproductive toxicant.

To verify that the germline nuclei were culled by apoptosis and not by necrotic death, we monitored the expression of the phagocytic receptor CED-1, which is activated during apoptotic corpse engulfment and creates a ring of expression around the apoptotic nuclei.^17^ We found an increased number of CED-1 positive nuclei (*i.e.* nuclei surrounded by CED-1 expression) in the germlines of worms exposed to Maneb and Diazinon when compared to DMSO (Supplemental Fig. S2†). Fenarimol, while showing a higher number of CED-1 positive nuclei compared to control, did not reach significance.

We also confirmed the germline nuclei are dying by apoptosis by examining the dependency of this process on the activity of the caspase CED-3, a well characterized mediator of the apoptotic pathway in the *C. elegans* germline.^18^ As mentioned above, wild-type worms exposed to all three pesticides show elevated acridine orange-positive nuclei in late pachytene. By contrast, worms homozygous for the strong loss-of-function allele *ced-3(n717)* showed a remarkable decrease in the number of acridine orange stained nuclei (Fig. 2A and 2D) indicating that the germline nuclear death following pesticide exposure is apoptotic in nature.

### Activation of the pachytene checkpoint in response to pesticide exposure

**Fig. 3:**
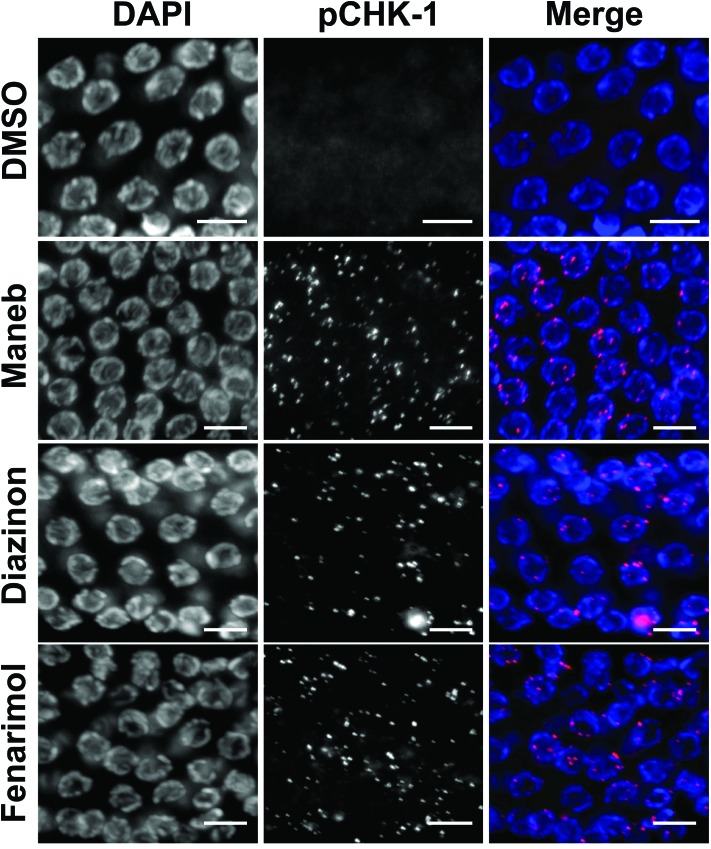
Exposure to Maneb, Diazinon and Fenarimol results in an activation of the DNA damage checkpoint. Representative immunostaining images for phosphorylated CHK-1(pCHK-1, red) in mid to late pachytene nuclei of dissected gonads exposed to either DMSO (0.1%) or Maned, Diazinon or Fenarimol exposed at 100 μM. *n* = 8–12. Scale bar, 5 μm.

The culling of defective germline nuclei by apoptosis is a well conserved process that relies on the coordinated activity of several key checkpoint components including the phosphorylation of the serine/threonine-specific protein kinase CHK-1 in response to DNA damage and unrepaired chromosomal recombination.^19,20^ Thus, we monitored the expression of phosphorylated CHK-1 (pCHK-1) in the germline of *C. elegans* following exposure to Maneb, Diazinon and Fenarimol. For all three pesticides, we observed a dramatic up-regulation of pCHK-1 expression on late pachytene germline nuclei (Fig. 3) indicating the activation of the recombination checkpoint.

### Alteration of the kinetics of meiotic recombination

**Fig. 4:**
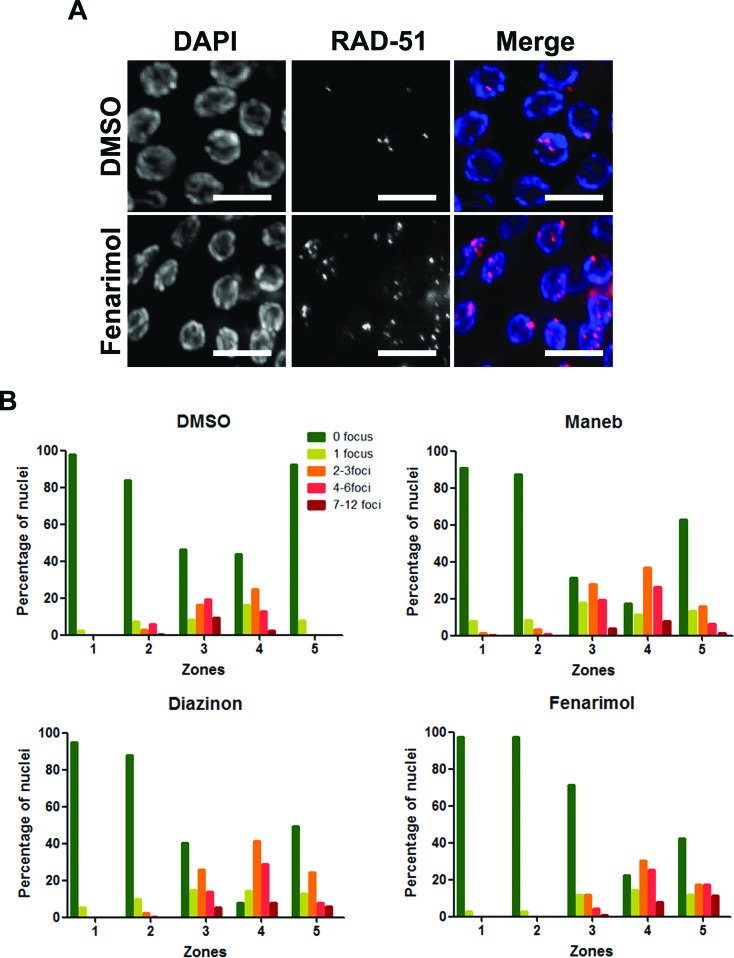
Exposure to Maneb, Diazinon and Fenarimol results in an alteration of the kinetics of meiotic recombination. (A) Mid-late pachytene nuclei from germlines exposed to either DMSO or a representative pesticide (Fenarimol). The pesticide-exposed germlines show elevated levels of RAD-51 (red) foci compared with control. Scale bar = 5 μm. (B) Sketch map showing how gonads were divided into 5 zones along the germline axis for analysis purposes. (C) Histograms depict the quantitation of RAD-51 foci performed in whole-mount gonads from age-matched hermaphrodites exposed to either DMSO, Meneb, Diazinon or Fenarimol. The number of RAD-51 foci per nucleus is categorized by the color code shown on the right. The percentage of nuclei observed for each category (*y* axis) is depicted for each zone (*x* axis). Zones 1, 2, 3 , 4 and 5 correspond to mitotic, transition, early, mid- and late pachytene stages respectively.

The activation of the DNA repair checkpoint in late pachytene could originate from the deregulation of the repair of the programmed double-strand breaks (DSBs) inherent to meiosis and leading to the production of incompletely resolved recombination events in nuclei that are then cleared by apoptosis. To test this hypothesis, we monitored the kinetics of expression of RAD-51, a central factor in the process of recombination, important for homologous strand invasion.^21^ As expected in control germlines, RAD-51 is not detected in germline nuclei undergoing mitosis and only appears as foci during the stage of pachytene. As more nuclei progress through pachytene and undergo recombination, the number of foci increases until it peaks around mid-pachytene. Then as recombination proceeds and eventually resolves, the number of RAD-51 foci gradually decreases to background levels by the end of pachytene/early diplotene.^22,23^ By contrast, we observed an altered kinetics of RAD-51 following exposure to the three pesticides where RAD-51 levels (Fig. 4). The effect was particularly pronounced for nuclei in mid-pachytene as well as in late pachytene (zones 4 and 5 respectively) which displayed a high number of RAD-51 foci when compared with DMSO control (Fig. 4). The alteration of RAD-51 kinetics was not driven by the presence of a few abnormal nuclei with very high levels of RAD-51 but instead was caused by a general delay in the removal of RAD-51 across the population of pachytene nuclei. These results suggest a profound alteration of the kinetics of DSBs repair following exposure to Maneb, Diazinon and Fenarimol.

The alteration of the kinetics of double-strand break repair may be at the root of the elevated levels of germline apoptosis (Fig. 2). To confirm that the generation and persistence of recombination intermediates following exposure cause an increase in apoptosis, we monitored apoptosis levels in two mutant backgrounds: *cep-1* and *spo-11*. SPO-11 is a protein related to topoisomerases and is universally required for the creation of DNA double-strand breaks used during homologous recombination. CEP-1, on the other hand, is the *C. elegans* homologue of p53 and its activity is necessary for the response to DNA damage and incomplete homologous recombination. In both *spo-11* and *cep-1* mutant backgrounds the increase in germline apoptosis following exposure to Maneb, Diazinon and Fenarimol when compared to control is completely abolished (ESI Fig. 3†). As apoptotic levels are very low when double strand breaks are not formed (*spo-11* mutant) or when they cannot be detected (*cep-1* mutant), we concluded that the induction of apoptosis in late pachytene following pesticide exposure is likely caused by the inability to repair endogenous breaks during meiosis.

As mentioned above, germ cell apoptosis can also be triggered by the synapsis checkpoint. We therefore monitored the establishment and maintenance of the synaptonemal complex (SC), a multi-protein structure that holds homologous chromosomes together and is required for stabilizing homologous chromosome interaction and for recombination.^24^ We did not observe evidence of incomplete synapsis or defective SC in exposed worms as tested by immunofluorescence for the SC-component SYP-1 (data not shown) suggesting that germ cell loss by apoptosis is likely to occur principally by activation of the DNA-damage/recombination checkpoint.

### The early meiotic impairments correlate with oogenesis defects

**Fig. 5:**
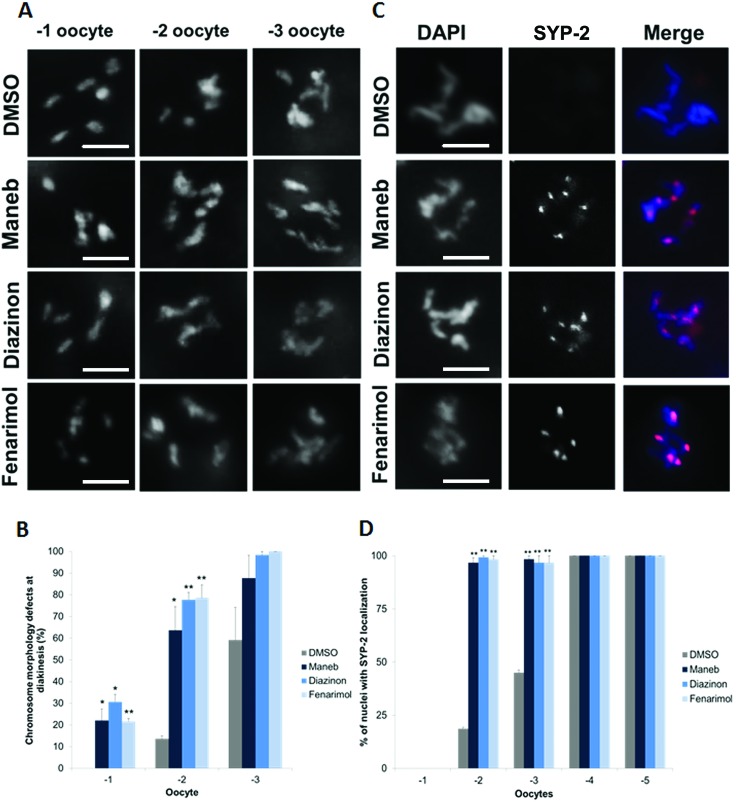
Exposure to Maneb, Diazinon and Fenarimol causes severe defects at diakinesis. (A) Six intact bivalents are observed in control (DMSO, 0.1%) -1 oocytes. By contrast, chromosomes with an frail, fragmented and uncondensed appearance are observed in –1 oocytes in Maneb (100 μM), Diazinon (100 μM) and Fenarimol (100 μM) exposed germlines. Scale bar, 5 μm. (B) Quantification of the chromosome morphology defects observed in bivalents at diakinesis (–1, –2 and –3 oocytes). Error bars represent SEM. *n* = 57–68, *p ≤ 0.05 and **p ≤ 0.005. (C) Immunocololalizaton of SYP-2 (red) on DAPI-stained (blue) –2 oocytes reveals a delay in SC disassembly and bialent morphogenesis in Maneb, Diazinon and Fenarimol exposed germlines. Scale bar, 5 μm. (D) Percentage of nuclei with expression of SYP-2 in –1 to –5 oocytes. Error bars represent SEM. *n* = 30, **p ≤ 0.005.

The defects observed at the early stages of meiosis prompted the examination of the effect on the later meiotic stages such as oocyte formation. In particular, we examined chromosome morphology at diakinesis, a stage at which chromosomes undergo further condensation in preparation for fertilization and metaphase I. For all three pesticides, we found evidence of chromosome condensation defects in the –1 and –2 oocytes, *i.e.* the two oocytes that the closest to the spermatheca. Compared to DMSO control where chromosomes assume a compact morphology, Maneb, Diazinon and Fenarimol treated worms display diakinetic chromosomes that are less condensed and carry a frail appearance (Fig. 5A, B). As these results suggested a delay in chromosome morphogenesis, we examined the kinetics of disassembly of the SC. Importantly, the step-wise disassembly of the SC is also crucial for subsequent chromosome segregation at Meiosis I.^25,26^ As such, control germlines show completion of SC disassembly in late diakinesis and absence of detectable SC on the chromosomes of the –1 and –2 oocytes (Fig. 5C, D). By contrast, we observed that the –2 oocytes of worms exposed to Maneb, Diazinon and Fenarimol retain SYP-2 expression indicating a delay in SC disassembly.

In rare instances, approximately 2% of exposed worms, following Diazinon and Maneb but not Fenarimol or DMSO exposure, we observed a significant reorganization of the meiotic stages. As shown in ESI Fig. 4,† we find evidence of germline nuclei with a pachytene-like morphology located at the proximal end of the germline where germ cells normally are in the stage of diakinesis. To our knowledge, such disorganization of the germline has not been previously reported either in the context of chemical exposures or in genetic experiments.

### Maneb, Diazinon and to a lesser extent Fenarimol exposure causes reduction in fertility

**Fig. 6:**
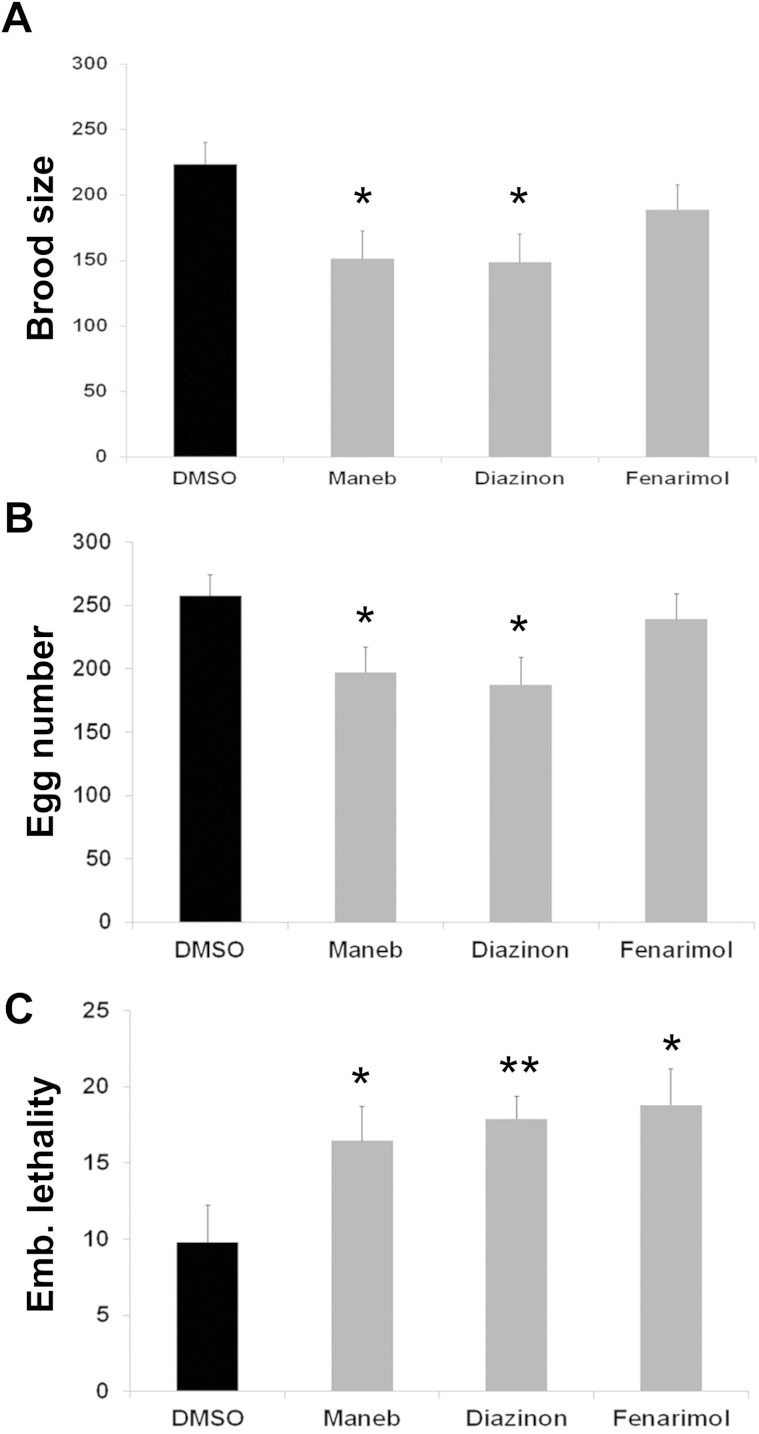
Exposure to Maneb, Diazinon and Fenarimol results fertility. (A) Brood size (total progeny number) from hermaphrodites exposed either to vehicle (0.1% DMSO) or to Maneb, Diazinon or Fenarimol (100 μM). Error bars represent SEM. *n* = 8; *p ≤ 0.05. (B) Fertilized egg number from hermaphrodites exposed either to vehicle (0.1% DMSO) or to Maneb, Diazinon or Fenarimol (100 μM). Error bars represent SEM. *n* = 8; *p ≤ 0.05. (C) Embryonic lethality of the progeny of hermaphrodites exposed either to vehicle (0.1% DMSO) or to Maneb, Diazinon or Fenarimol (100 μM). Error bars represent SEM. *n* = 8; *p ≤ 0.05 and **p ≤ 0.005.

To investigate the consequence of exposure to the 3 pesticides on fertility, we quantified the number and viability of the exposed worm's progeny. We observed that exposure to Maneb, Diazinon and Fenarimol, decreased the total number of progeny (number of surviving adult progeny) by as much as 30% for Maneb and Diazinon (*p* ≤ 0.05) while Fenarimol showed a trend towards decrease but did not reach significance (Fig. 6A). Further analysis revealed that the reduction in progeny mostly originated from a decrease in fertilized egg numbers compounded by a decrease in embryonic viability, both phenotypes commonly observed following disruption of meiosis (Fig. 6B and 6C). Therefore, Maneb and Diazinon exposures are associated with a steep decrease in fertility in *C. elegans*, while Fenarimol's reduction in fertility appears less pronounced.

## Conclusions

In the present study, we have shown that exposure to three pesticides, Maneb, Diazinon and Fenarimol, previously identified in an assay for aneugenicity, each leads to acute germline nuclear loss in the nematode *C. elegans*. By monitoring apoptosis following exposure, we showed that this germline nuclear loss results from activation of the pCHK-1 dependent pachytene checkpoint and induction of apoptosis. The observed germ cell death is likely the result of defects in the meiotic recombination process as evidenced by alteration of the kinetics of the recombination marker RAD-51 and the dependency of the apoptotic phenotype on the activity of SPO-11 and CEP-1. These early meiotic impairments are likely at the root of the chromosome condensation defects observed at later stages of meiosis in oocytes and also of the increased embryonic lethality. The defects in completion of homologous recombination, necessary for subsequent chromosome division at metaphase I, and in chromosome morphogenesis at the end of Prophase I following exposure to Maneb, Diazinon and Fenarimol provide an explanation for the previously reported increase in embryonic aneuploidy.^2^

While we initially considered four pesticides, one of them, Triflumizole did not show overt germ cell loss or apoptosis despite being highly aneugenic (12-fold over DMSO).^2^ This suggested that Triflumizole may cause embryonic aneuploidy by directly impacting the early embryonic divisions and not by altering germline processes. By contrast, Maneb, Diazinon and Fenarimol all caused similar effects on the germline including disrupting the proper kinetics of double-strand break repair. All 3 chemicals show a normal mitotic zone and normal entry into meiosis based on nuclear morphology and synaptonemal complex staining. However, they show induction of apoptosis in late pachytene, likely mediated by the generation of aberrant recombination intermediate or the inability to repair them. The exact mechanism of action of these compounds remains to be elucidated but our results suggest that the molecular events upstream of RAD-51 turnover are altered. Ongoing work on the alteration of gene expression through RNA-seq analysis will likely reveal the impacted step. The similarities of the effect of exposure to Maneb, Diazinon and Fenarimol suggest that the process of double-strand break repair during meiosis may be a common target of germline toxicants, especially aneugens. The common effect of these three pesticides may originate from the screen that was used to identify them as aneugens. Indeed, the *Pxol-1::GFP* reporter strain has been previously used in a genetic context for the isolation of genes important for meiosis which identified the MutS homologue *msh*-5, a factor required for proper completion of homologous recombination and cross-over formation. We cannot exclude however the possibility that altered homologous recombination may be a reflection of germline toxicity as opposed to being the cause of the other observed germline phenotypes. Thus meiotic recombination and RAD-51 kinetics in particular would be a biomarker of germline toxicity. Nonetheless, it is likely that meiotic double strand break repair is a common target of germline toxicants as this process has been shown to be particularly sensitive to a number of environmental compounds including carcinogens, leading to abnormal DNA repair and genomic instability (reviewed in ref. 27).

Some meiotic defects were unexpected including the disorganization of progression through meiosis observed in Maneb and Diazinon-exposed worms. Although rare in frequency, the extent of rearrangement of the order of the germline nuclei is profound and manifests by the presence of pachytene-like nuclei located at the proximal end of the gonad when the nuclei are normally in diakinesis and have been cellularized. The low occurrence of these abnormal gonads did not allow us to capture them in experiments where the germline was stained for the synaptonemal component SYP-1. Therefore, we have been unable to confirm that these nuclei, beyond their pachytene morphology, also display evidence of synapsis typical of nuclei in pachytene. However, the presence of DNA “tracks” reminiscent of synapsed homologous chromosomes characteristic of pachytene nuclei.^25^ We hypothesize that these nuclei may result from the incomplete differentiation of germline nuclei that remain in a pachytene state while spatially progressing along the distal-proximal gonadal axis. This would therefore represent a unique dissociation of the spatial and differentiation gradient of germline nuclei characteristic of the *C. elegans* germline.

Our results indicate that out of the four pesticides tested Maneb, Diazinon and Fenarimol all display the hallmarks of reproductive toxicity in *C. elegans*. This data is consistent with the fertility assays published as part of the ToxRef database program which tested more than 200 chemicals for their reproductive and multigenerational effects.^9^ In particular, Diazinon has been shown to cause a decrease in litter size, fertility as well as decreased implantation rates and abnormal ovarian morphology in rats. Maneb only causes ovarian defects while Fenarimol only causes a decrease in fertility. However, the data presented here suggests a profound effect on the meiotic program for all three pesticides manifested by an acute loss of germ cells and a dramatic alteration of the meiotic double strand break repair process.

In conclusion, this study highlights some of the advantages of using small animal models to examine the impact of chemical exposure on complex cellular and developmental processes. In particular, we have chosen the model *C. elegans* as it allows for the fast analysis of the evolutionarily conserved molecular events guiding early germ cell differentiation. By contrast, in female mammals, the same meiotic stages observed here take place during embryogenesis and are therefore difficult to assess. Through a series of expression and mechanistic analyses, we have been able to determine the steps of meiotic differentiation that are affected by exposure to three pesticides: Maneb, Diazinon and Fenarimol. This study not only offers insights on the impact of these chemicals on the germline in *C. elegans* but also provides a rationale for the prioritization of these chemicals for further study of their reproductive toxicity in rodent models and in humans, especially with regards to their effect on early germ cell development.

## Experimental

### *C. elegans* strains and growth conditions

*C. elegans* strains were cultured as described in^28^ on nematode growth medium (NGM) plates seeded with *E. coli* (strain OP50) at 20 °C. The N2 Bristol strain was used as the wild-type strain. The following mutations and chromosome rearrangements were used in this study: LGI: [Pced-1::ced-1::gfp; unc-76]; *cep-1(lg12501)* and LGIV: *spo-11(ok79); ced-3(n717)*.

### Drug exposures

The pesticides Maneb, Diazinon and Fenarimol were purchased from Sigma-Aldrich (St. Louis, MO) were dissolved in dimethyl sulfoxide (DMSO; 0.1 M) prior to exposure. Gravid nematodes were lysed in an alkaline hypochlorite solution in order to generate age-matched embryos synchronized populations.^29^ The embryos were cultured on 10-cm NGM plates without bacteria for 1 day at 20 °C to generate a large pool of synchronized L1-stage worms. The L1-stage worm population was transferred to 10-cm NGM plates seeded with OP50 bacteria and cultured for 65 h at 15 °C to generate a large pool of L4-stage worms. After quantification under the microscope of the number of worms in population samples, 500 worms were transferred to 2 mL tubes with M9 buffer with bacteria, to which the pesticides were subsequently added at a final concentration of 100 μM. Each experiment contained a negative control of 0.1% DMSO. The worms were incubated with shaking on a nutator for 24 h at 20 °C. After the incubation the synchronized population of gravid adult worms settled by gravity and washed with M9 buffer before using it for further analysis.

### Immunostaining

Germlines were dissected from young adults and frozen on a block at –80 °C for 2 minutes, then placed in methanol at –20 °C for 1 min followed by a fixation with 4% formaldehyde for 30 min at room temperature and blocked in PBST (PBS with 0.1% Tween 20) and 0.5% BSA for 1 h at room temperature. Samples were incubated with the primary antibody diluted n PBST overnight at 4 °C followed by incubation with the secondary antibody diluted in PBST for 2 h at RT. Primary antibodies were used at the following dilutions: goat α-SYP-1, 1 : 100; rabbit α-SYP-2, 1 : 100; rabbit α-RAD-51, 1 : 8000; goat α-pCHK-1, 1 : 50 (Santa Cruz Biotechnology)Secondary antibodies used were Cy3 anti-rabbit, 1 : 700 (Jackson Immunochemicals), Cy3 anti-goat, 1 : 500 (Jackson Immunochemicals).

### Time course analysis for RAD-51 foci

The germlines were divided in five zones starting with the mitotic zone and the number of foci per nucleus was counted for each of the five zones of the germline as previously described.^23^ Six germlines were scored for each exposure group. The average numbers of nuclei scored per zone (*n*) for DMSO- and pesticide-exposed *N2* wild-type worms were as follows: zone 1, *n* = 57; zone 2, *n* = 58; zone 3, *n* = 50; zone 4, *n* = 48; zone 5, *n* = 36.

### Assessment of reproduction

Reproductive ability was measured by monitoring embryonic lethality, larval lethality and brood size. To assess embryonic lethality, the number of L1 larvae was divided by the number of embryos produced. To assess larval lethality, the number of adult worms was divided by the number of L1 larvae. Finally, to assess brood size, the total number of surviving adults was counted. Ten nematodes were examined for each exposure group and control.

### Germ cell apoptosis assay

After the 24 h exposure, worms were placed into 0.4 ml acridine orange staining solution with bacteria, kept in the dark for 2 h, then transferred to a new plate and scored after 30 min under a fluorescence microscope. Apoptotic corpses stained with acridine orange were counted in one gonad arm per worm.

For CED-1::GFP analysis, worms from the strain ZH814 were exposed as described above and directly visualized under fluorescence following exposure as previously reported.^30^

### Imaging and microscopy

Immunofluorescence and DIC images were collected at 0.2 μm intervals with a Nikon Eclipse Ni microscope and a Photometrics Cool SNAP HQ^2^ camera. Images were subjected to deconvolution analysis using the NIS Elements program (version AR 4.02.01).

### Statistical analysis

Data are presented as means ± SEM. All statistical analyses were performed using a two-tailed unpaired Student's test with unequal variance. Statistical significance was defined as *p* ≤ 0.05.
