# Supplementary material for: Reproductive toxicity and meiotic dysfunction following exposure to the pesticides Maneb, Diazinon and Fenarimol
Source: Toxicol Res (Camb). 2015 Feb 2;4(3):645–54. doi: 10.1039/c4tx00141a (PMC4433152; doi:10.1039/c4tx00141a)

Electronic Supplementary Material (ESI) for Toxicology Research.
This journal is © The Royal Society of Chemistry 2015

Supplemental Figure 1

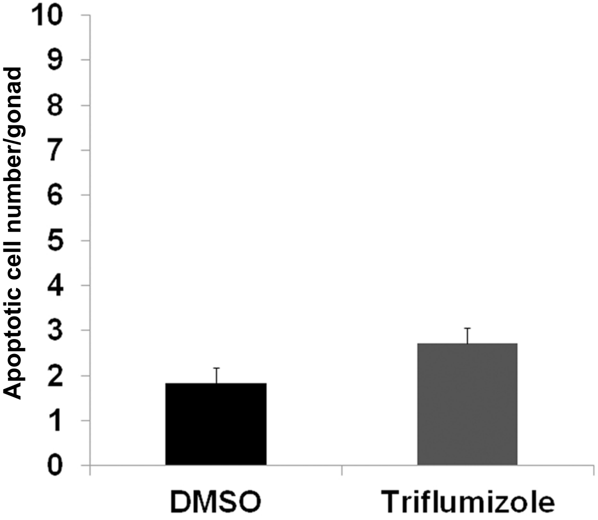

Supplemental Figure 2

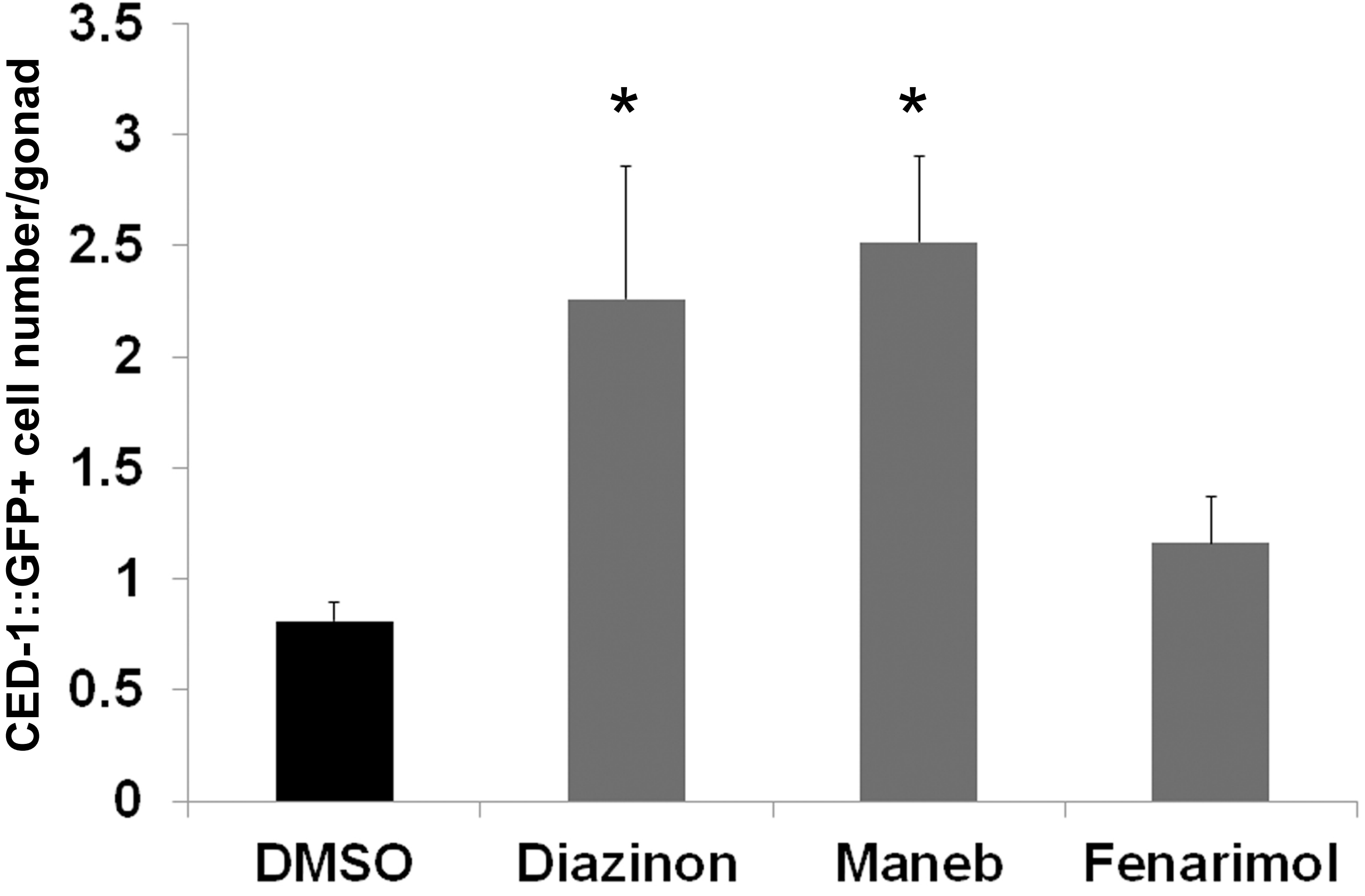

Supplemental Figure 3

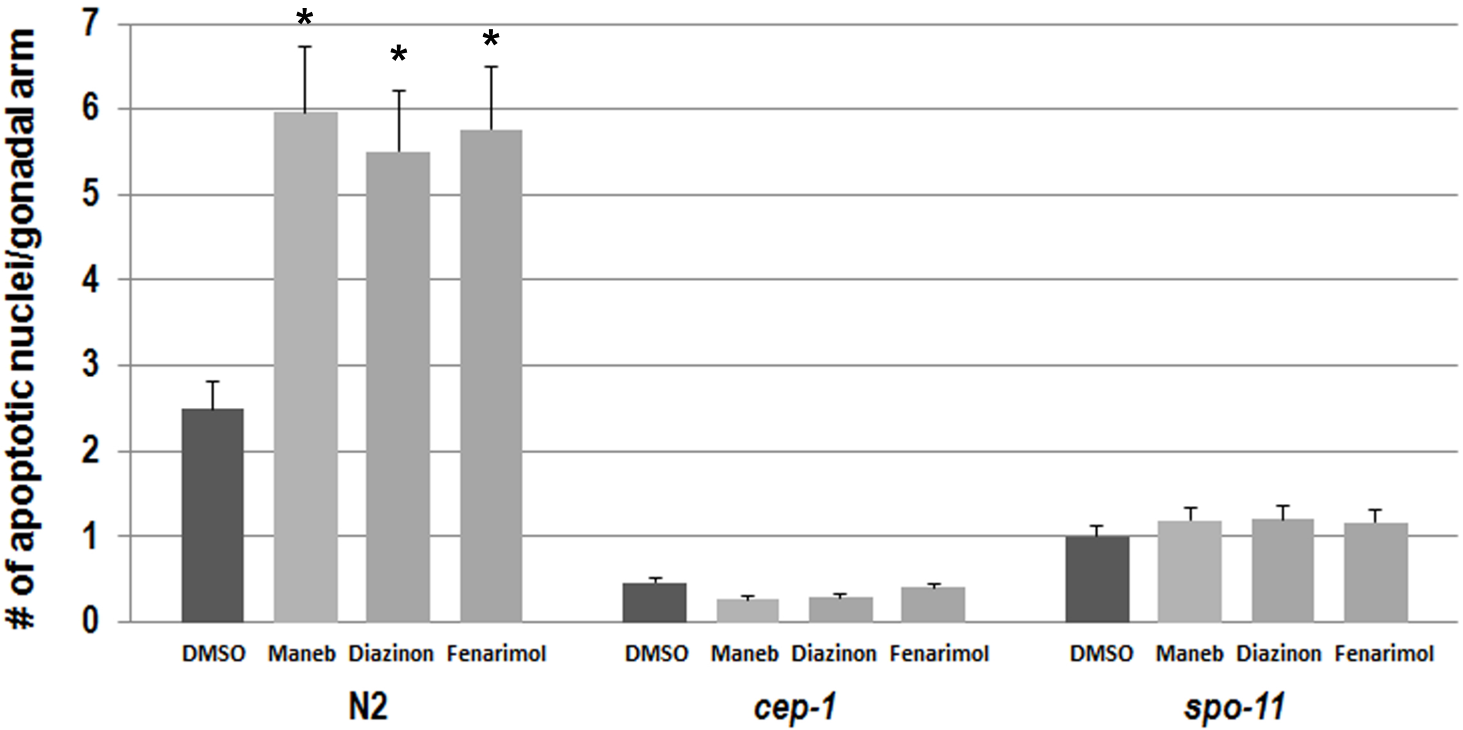

Supplemental Figure 4

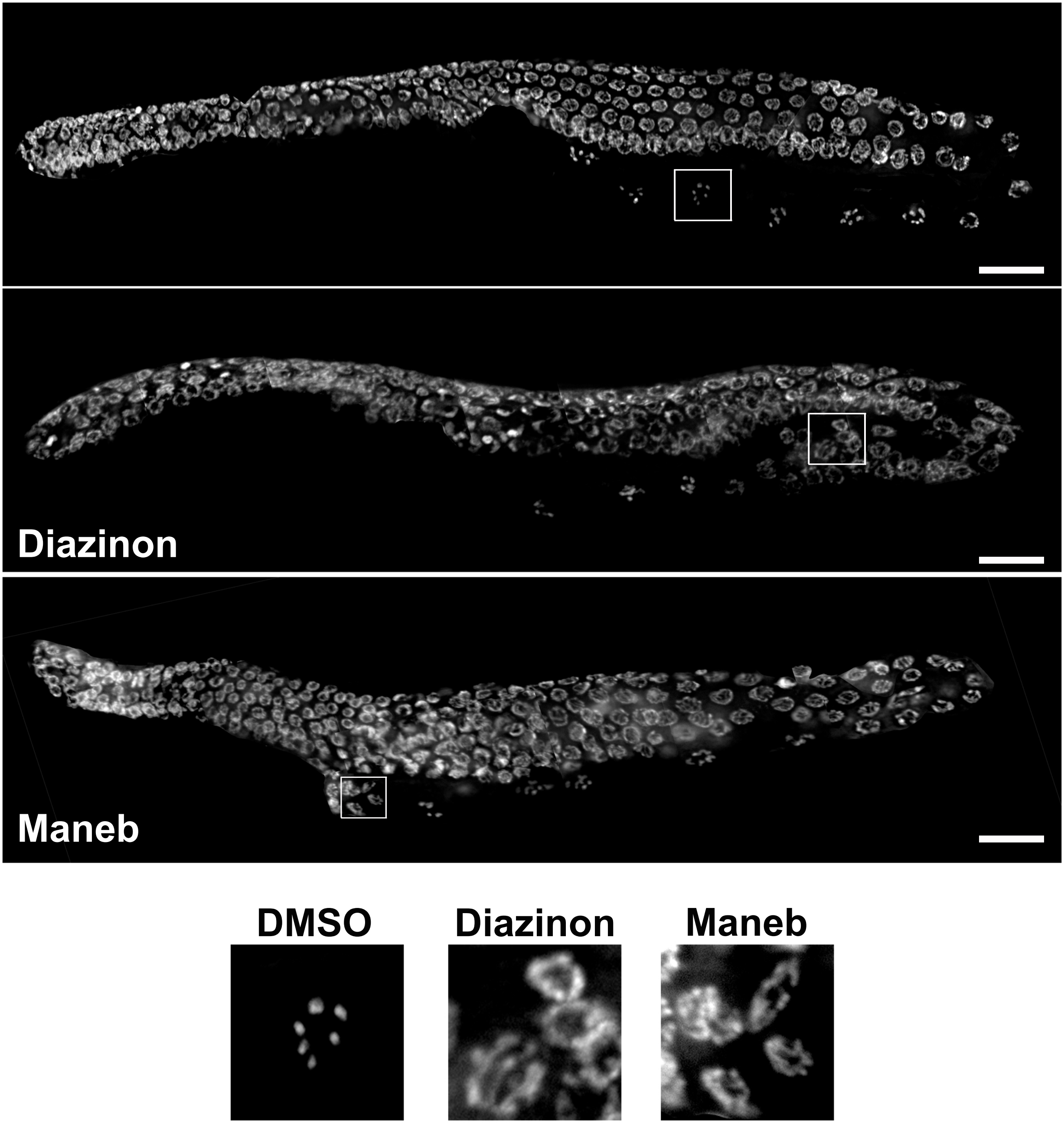

Supplement: Supplementary file 2 [file TX-004-C4TX00141A-s002.pdf]
