# Supplementary material for: Reproductive toxicity and meiotic dysfunction following exposure to the pesticides Maneb, Diazinon and Fenarimol
Source: Toxicol Res (Camb). 2015 Feb 2;4(3):645–54. doi: 10.1039/c4tx00141a (PMC4433152; doi:10.1039/c4tx00141a)
Supplement: Supplementary file 3 [file TX-004-C4TX00141A-s003.pdf]

Electronic Supplementary Material (ESI) for Toxicology Research.
This journal is © The Royal Society of Chemistry 2015

Supplemental Information

Supplemental Figure Legends

Supplemental Figure 1: Exposure to Triflumizole does not cause germline apoptosis

Quantification of germ cell apoptosis in wild type N2 worms exposed either with vehicle (0.1% DMSO) or Triflumizole (100µM) for 24 hours. Error bars represent SEM. n = 105-81.

Supplemental Figure 2: Quantification of CED-1::GFP-positive nuclei following exposures to DMSO or pesticides.

Quantification of the number of apoptotic cells as visualized by CED-1::GFP staining and corrected for the total number of cells at pachytene. Diazinon and Maneb exposure reached significance while Fenarimol showed a modest increase. * $p \leq 0.05$.

Supplemental Figure 3: Quantification of apoptotic levels following exposures to DMSO or pesticides in wild type (N2), *spo-11* and *cep-1* mutant backgrounds

Quantification of the number of apoptotic cells as visualized by acridine orange staining in N2 and *spo-11* and *cep-1* mutants. While Diazinon, Maneb and Fenarimol all caused a significant increase in apoptosis levels, this increase was abolished in *cep-1* and *spo-11* mutants. * $p \leq 0.05$.

Supplemental Figure 4: Disorganization of meiotic differentiation

Picture of a full gonadal arm of worms exposed to DMSO, Diazinon and Maneb. A profound disorganization of the germline is evident and includes the presence of nuclei resembling those at pachytene (insets). In particular, the presence of DNA “tracks” suggesting homologous synapsis is observed in several nuclei located in diakinesis. Scale bar, 15 μm.
